# De novo mutations in childhood cases of sudden unexplained death that disrupt intracellular Ca^2+^ regulation

**DOI:** 10.1073/pnas.2115140118

**Published:** 2021-12-20

**Authors:** Matthew Halvorsen, Laura Gould, Xiaohan Wang, Gariel Grant, Raquel Moya, Rachel Rabin, Michael J. Ackerman, David J. Tester, Peter T. Lin, John G. Pappas, Matthew T. Maurano, David B. Goldstein, Richard W. Tsien, Orrin Devinsky

**Affiliations:** ^a^Department of Genetics, University of North Carolina at Chapel Hill, Chapel Hill, NC 27599;; ^b^Institute for Genomic Medicine at Vagelos College of Physicians and Surgeons, Columbia University Irving Medical Center, New York, NY 10032;; ^c^Comprehensive Epilepsy Center, New York University Grossman School of Medicine, New York, NY 10016;; ^d^Sudden Unexplained Death in Childhood Foundation, Roseland, NJ 07068;; ^e^Neuroscience Institute, New York University Grossman School of Medicine, New York University, New York, NY 10016;; ^f^Department of Neuroscience and Physiology, New York University, New York, NY 10016;; ^g^Institute for Systems Genetics, New York University Grossman School of Medicine, New York, NY 10016;; ^h^Department of Pediatrics, New York University Grossman School of Medicine, New York, NY 10016;; ^i^Division of Heart Rhythm Services, Department of Cardiovascular Medicine, Windland Smith Rice Genetic Heart Rhythm Clinic and Windland Smith Rice Sudden Death Genomics Laboratory, Mayo Clinic, Rochester, MN 55902;; ^j^Division of Pediatric Cardiology, Department of Pediatric and Adolescent Medicine, Windland Smith Rice Genetic Heart Rhythm Clinic and Windland Smith Rice Sudden Death Genomics Laboratory, Mayo Clinic, Rochester, MN 55902;; ^k^Department of Molecular Pharmacology & Experimental Therapeutics, Windland Smith Rice Genetic Heart Rhythm Clinic and Windland Smith Rice Sudden Death Genomics Laboratory, Mayo Clinic, Rochester, MN 55902;; ^l^Department of Laboratory Medicine and Pathology, Mayo Clinic, Rochester, MN 55902

**Keywords:** sudden death in children, cardiac arrhythmia, seizure disorder, genetics, calcium signaling

## Abstract

Sudden unexplained death in childhood (SUDC) is an understudied problem. Whole-exome sequence data from 124 “trios” (decedent child, living parents) was used to test for excessive de novo mutations (DNMs) in genes involved in cardiac arrhythmias, epilepsy, and other disorders. Among decedents, nonsynonymous DNMs were enriched in genes associated with cardiac and seizure disorders relative to controls (odds ratio = 9.76, *P* = 2.15 × 10^−4^). We also found evidence for overtransmission of loss-of-function (LoF) or previously reported pathogenic variants in these same genes from heterozygous carrier parents (11 of 14 transmitted, *P* = 0.03). We identified a total of 11 SUDC proband genotypes (7 de novo, 1 transmitted parental mosaic, 2 transmitted parental heterozygous, and 1 compound heterozygous) as pathogenic and likely contributory to death, a genetic finding in 8.9% of our cohort. Two genes had recurrent missense DNMs, *RYR2* and *CACNA1C*. Both *RYR2* mutations are pathogenic (*P* = 1.7 × 10^−7^) and were previously studied in mouse models. Both *CACNA1C* mutations lie within a 104-nt exon (*P* = 1.0 × 10^−7^) and result in slowed L-type calcium channel inactivation and lower current density. In total, six pathogenic DNMs can alter calcium-related regulation of cardiomyocyte and neuronal excitability at a submembrane junction, suggesting a pathway conferring susceptibility to sudden death. There was a trend for excess LoF mutations in LoF intolerant genes, where ≥1 nonhealthy sample in denovo-db has a similar variant (odds ratio = 6.73, *P* = 0.02); additional uncharacterized genetic causes of sudden death in children might be discovered with larger cohorts.

## Significance

Approximately 400 United States children 1 y of age and older die suddenly from unexplained causes annually. We studied whole-exome sequence data from 124 “trios” (decedent child and living parents) to identify genetic risk factors. Nonsynonymous mutations, mostly de novo (present in child but absent in both biological parents), were highly enriched in genes associated with cardiac and seizure disorders relative to controls, and contributed to 9% of deaths. We found significant overtransmission of loss-of-function or pathogenic missense variants in cardiac and seizure disorder genes. Most pathogenic variants were de novo in origin, highlighting the importance of trio studies. Many of these pathogenic de novo mutations altered a protein network regulating calcium-related excitability at submembrane junctions in cardiomyocytes and neurons.

Sudden unexplained death affects all ages and socioeconomic groups. Prevention focuses on reducing environmental causes (e.g., unsafe sleep environment in infants), treating medical conditions (e.g., sleep apnea) and identifying high-risk individuals (e.g., young athletes with exertional syncope). When medical record review, death scene investigation, autopsy, and toxicology provide no definite cause of death, decedent DNA sequencing may identify pathogenic variants that confer susceptibility for lethal ventricular arrhythmias (1, 2). Sudden cardiac death series in children and young adults with unrevealing autopsies found pathogenic cardiac gene variants in 25% of cases (1, 3). By contrast, among children ages 1 to 10 y who died in sleep—most sudden unexplained deaths in childhood (SUDC)—pathogenic cardiac gene variants occurred in only 4.2% of cases (3).

Most SUDC cases affect 1- to 4-y-olds, for whom “undetermined/unexplained” is the fifth leading category of death (4, 5). These cases challenge the medico-legal death investigation systems, which often lack standardized methods and resources (6). These limitations and the perceived need to identify a cause of death (6, 7) lead to some unexplained pediatric deaths being misclassified as “explained” (e.g., mild respiratory illness) (8), which likely underestimates the true incidence of unexplained childhood deaths. Although sudden death studies focus on cardiac genes (9), seizures likely cause some SUDC since febrile seizures occur in 29 to 32% of decedents, ∼10-fold greater than the general population (10–12). This suggests a contributory mechanism similar to sudden unexpected death in epilepsy (SUDEP) (7).

In comparison to the 1,400 annual United States cases of sudden infant death syndrome (SIDS), there are 400 SUDC cases. However, SIDS has received >$500 million for research and public health education in the United States (13), with >12,000 articles in PubMed, while SUDC has received no targeted research funding and <45 articles in PubMed. Thus, there has been a 300:1 disparity in funding and publications for two lethal childhood disorders with a 4:1 incidence. Despite the wealth of SIDS research, a recent review of genetic findings identified only four pathogenic de novo mutations (DNMs), which are present in the proband and absent in both parents. A major hindrance is that molecular autopsies typically lack parental data, limiting identification of DNMs.

To identify potential pathogenic de novo or inherited coding single nucleotide variants and indels in SUDC, we studied genetic data from 124 decedent/parental trios, the largest trio dataset of any sudden pediatric death cohort. We sought to identify de novo and inherited pathogenic variants, focusing initially on cardiac and epilepsy genes given prior epidemiological and genetic studies (14, 15). We also functionally characterized pathogenic variants involving overlapping pathways to assess the role of impaired cardiac/neuronal Ca^2+^ signaling and excitability as a common pathogenic mechanism. All cases were obtained from the SUDC Registry and Research Collaborative (SUDCRRC), a registry approved by the New York University Institutional Review board (#S14-01061), that supports the epidemiology of sudden death in children after age 1 y as occurring primarily in toddlers (5).

## Results

### Overall Characteristics of Our SUDC Cohort.

**Fig. 1.**
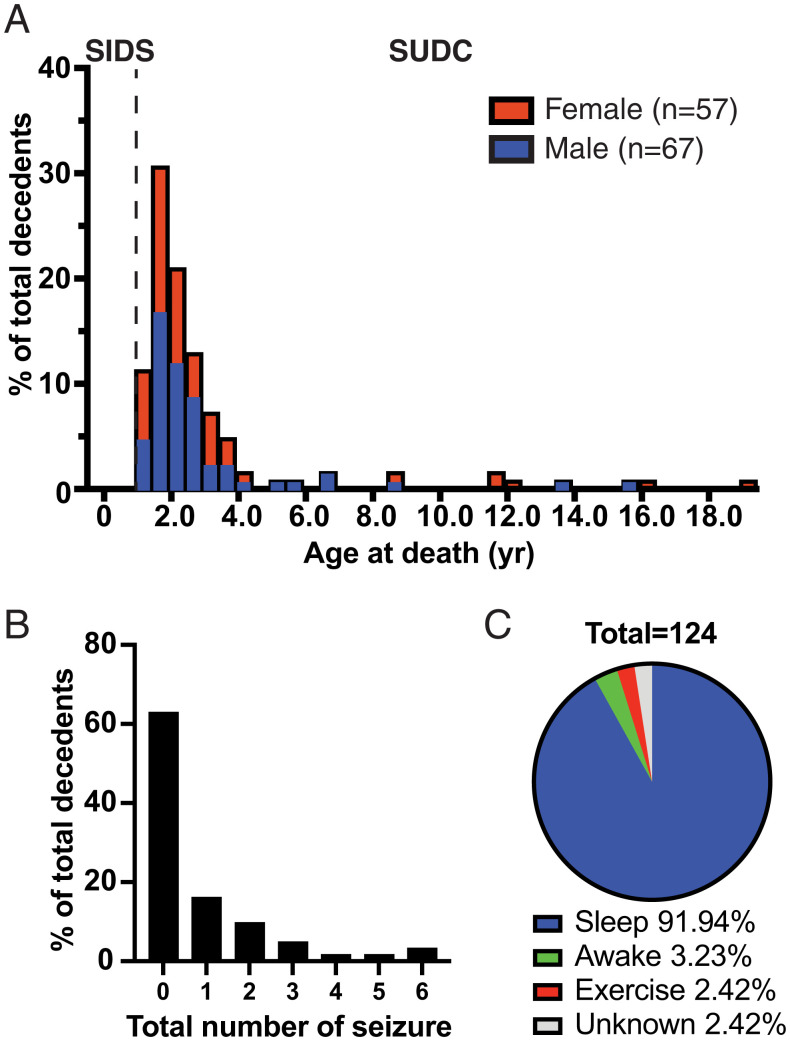
Overview of our SUDCRRC cohort. (*A*) Age and gender distribution of the decedents in our SUDCRRC cohort. We collected information of 124 decedents from 11 mo to 228 mo. (*B*) A histogram showing the number of total seizures the decedents had prior to death. (*C*) A pie chart showing the activities of the decedents at death. The majority of decedents were found dead during sleep.

Our 124 cases died at an average age of 34.2 ± 37.2 (SD) months (Fig. 1*A*, and *SI Appendix*, Fig. S1), consistent with the distribution of deceased ages in Centers for Disease Control and Prevention (CDC) WONDER data (5). Family histories are summarized in Datasets S1 and S2. Males comprised 54% of the cohort. Around 81.5% of the included cases were white. Gestational age averaged 38.8 ± 1.8 (SD) weeks. Medical records and family interviews revealed normal developmental milestones in 84.7%, seizures in 37.1% (Fig. 1*B*) (96% were febrile seizures), respiratory disorders (reactive airway disease, upper respiratory infections, laryngomalacia, or obstructive sleep apnea) in 21.8%, and minor cardiac congenital defects in 3.2%. Deaths were witnessed in 8.1% of cases; 91.2% died during apparent sleep (Fig. 1*C*).

Pathologists initially classified 40 cases as explained causes of death, including seizures (7), cardiac dysrhythmia/cardiomyopathy (11), infection (16), congenital anomalies (1), anoxic brain injury (3), and dehydration (1). However, independent reviews of the autopsy reports by a study forensic pathologist (P.T.L.) and principal investigator (O.D.) reclassified these 40 cases as undetermined due to lack of evidence.

Whole-exome sequencing was conducted on DNA from the decedent child and living parents. DNA was extracted from parent blood samples and child decedent blood (or postmortem tissue samples when blood was unavailable). Samples generated from cases and their living parents were all processed on the same sequence alignment and variant calling pipeline. From these processed data, pairwise relatedness measurements were produced and assessed to confirm parent/child relationships were concordant with reported family structure, and that family samples lacked cryptic relatedness with other trios or internal controls utilized (*SI Appendix*, *Supplementary Methods*).

### DNM Screen.

We identified 126 DNM calls in 124 SUDC trios. Sanger sequencing failed to validate one mutation that we have excluded, resulting in 125 DNM calls. The final DNM call set (Dataset S3) included 116 single nucleotide variants and 9 indels (∼1 coding single nucleotide variant and ∼0.1 coding indel per sample).

### Transmitted Postzygotic Mutation Screen.

We also identified five parental mosaic variant calls (Dataset S4); all were single nucleotide variants. Combining the mosaic variants with our DNM set, 130 calls were identified, 3.8% being mosaic in origin. This is slightly lower than 6.8%, the reported rate at which probands inherit heterozygous variants from a mosaic parent in a larger study (17). In our subsequent analyses, mosaic variants were only included in comparisons with internal controls, since with these samples we were able to conduct trio-based transmitted mosaic variant detection using the same approach.

### DNM Burden Relative to Expectation and Control Cohorts.

**Fig. 2.**
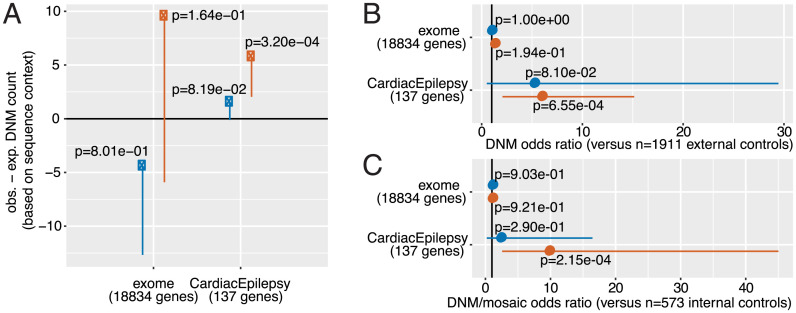
Observed versus expected DNM rates, partitioned by comparison type and annotation. (*A*) Observed minus expected mutation count across annotations (synonymous, in blue; nonsynonymous, in orange) across the exome and within 137 specifically selected genes whose dysfunction is reported to lead to a cardiac or seizure disorder that greatly increases risk of sudden death. Both estimates (dots) and 95% confidence interval lower bounds (lines) are provided, along with one-sided Poisson test *P* values. An observed minus expected DNM count significantly above zero suggests an overburden of variants in our cohort compared to a null expectation. (*B*) Results from two-sided Fisher exact tests of association between carrying at least one qualifying DNM and SUDC status, in 124 SUDC cases versus 1,911 external controls from Iossifov et al. (19). Mosaic variants excluded from comparisons with external controls. Odds ratio estimates (dots) and 95% confidence intervals (lines) are provided along with *P* values. (*C*) Results from two-sided Fisher exact tests of association between DNM carrier status and SUDC status, in this case using 573 internal control trios that are sudden death-negative. Mosaic variants included in comparison with internal controls since these samples underwent the exact same variant calling procedure as cases. Again, odds ratio estimates (dots) and 95% confidence intervals are provided along with *P* values.

We analyzed the burden of synonymous and nonsynonymous DNMs relative to expected mutation rate per gene (Fig. 2*A*), and relative to the load of similar mutations in non-SUDC trios (Fig. 2 *B* and *C*). The expected mutation rates were defined according to tables provided in the denovolyzeR package for R (18), and were computed for each combination of gene/annotation accounting for gene length and sequence context based on empirical mutation rates computed for sequence trimers in independent healthy trio data. For case/control comparisons, two sets of control trios were utilized. The first was a set of 1,911 previously published healthy control trios (19). The second was a set of 573 internal trios that were diagnosed with obsessive-compulsive disorder but without any reported overlapping sudden-death outcome. We included transmitted parental-mosaic variation in our case/control comparison versus internal control trios since we were able to call these variants in exactly the same way for these samples.

Comparing the synonymous and nonsynonymous DNM burden across the exome, we found insufficient evidence for a global excess of nonsynonymous mutations in the SUDC cohort. In rate-based and case/control comparisons, there was no excess of presumed neutral synonymous mutation in the SUDC trios [*P* = 0.801 for synonymous mutation rate in observed vs. expected analysis (Fig. 2*A*, left blue dot); *P* = 1.00 and 0.903 for synonymous mutation rate in case vs. external control and case vs. internal control analyses, respectively (Fig. 2 *B* and *C*, blue dots from the upper half of each panel)]. This suggests that our call set does not display an inflated mutation call rate and is suitable for similar comparisons of “functional” nonsynonymous mutation rate. There was no significant difference in nonsynonymous mutation rate versus expected mutation rate across the exome (observed = 95, expected = 85.5, obs.-exp. DNM count = 9.5, *P* = 0.164) (Fig. 2*A*, left orange dot). This result held in comparisons against external control trios (odds ratio = 1.27, *P* = 0.19) and internal control trios (odds ratio = 1.03, *P* = 0.92).

We then tested for evidence of enriched mutations in genes associated with cardiac or seizure disorders, both of which can trigger sudden death. We defined this set of genes (*n* = 137) (Dataset S5) based on genetic evidence for association with either sudden cardiac death (*n* = 95) (20) or epilepsy pathogenesis (*n* = 43) (21). In all tests, there was no evidence (*P* < 0.05) for excess of synonymous mutation in cases within these genes relative to expectation (Fig. 2). However, there was a highly significant elevation of nonsynonymous DNMs relative to expected mutation rate (observed = 7, expected = 1.25, obs.-exp. DNM count = 5.75, *P* = 3.2 × 10^−4^) (Fig. 2*A*, right orange dot). Consistent with this, SUDC cases carried a substantially higher burden of these mutations than both external control trios (odds ratio = 5.95, *P* = 6.55 × 10^−4^) (Fig. 2*B*) and internal control trios (odds ratio = 9.76, *P* = 2.15 × 10^−4^) (Fig. 2*C*). DNM rate is not substantially influenced by ancestry (22), and consistent with this, the case burden was still present in a European-ancestry comparison against internal controls (odds ratio = 7.60, *P* = 1.52 × 10^−3^).

To identify pathogenic de novo and transmitted parental mosaic variants within this cohort, we applied standard clinical genetic screens for 59 disease susceptibility genes proposed by the American College of Medical Genetics and Genomics (ACMG) (23). We found that 3 of 124 cases carried a nonsynonymous de novo or mosaic variant within one of these 59 genes. All three overlapping mutations (*RYR2* ×2, *TNNI3* ×1) impacted ACMG genes in the CardiacEpilepsy geneset. There was an excess of nonsynonymous mutations relative to expected rate (observed = 3, expected = 0.57, *P* = 0.01) and to internal control trios (odds ratio = 14.1, *P* = 0.02). However, the yield from the ACMG gene set was lower than from our CardiacEpilepsy gene set, suggesting that a standard ACMG screen is not optimal for genetic autopsy. Comparing the burden of ClinVar “pathogenic” or “likely pathogenic” missense variants in cases to internal control trios, an excess in SUDC probands was again evident (5 of 124 cases versus 2 of 573 controls, odds ratio = 11.9, *P* = 2.57 × 10^−3^); all case missense variants were in the CardiacEpilepsy geneset.

We classified a total of seven DNMs and one transmitted parental-mosaic variant as pathogenic or likely pathogenic within the context of this study (Table 1). Across our 124 trios this meant a yield of 5.6% and 0.8%, respectively, for a total yield of around 6.5%.

**Table 1.** Missense de novo and parental mosaic variants within genes whose dysfunction has previously been implicated with epilepsy or a potentially lethal cardiac channelopathy/cardiomyopathy

Var_ID_GRCh37	Proband_ID	Var_Type	Gene_Name	HGVS_c	HGVS_p	ClinVar_ClinSig	ClinVar_rsID
12-2613692-G-A	SUDCRRC-045	De novo	*CACNA1C*	c.1204G > A	p.Gly402Ser	Pathogenic	rs587782933
12-2613674-G-C	SUDCRRC-114	De novo	*CACNA1C*	c.1186G > C	p.Val396Leu	NA	NA
14-90870730-A-G	SUDCRRC-001	De novo	*CALM1*	c.293A > G	p.Asn98Ser	Pathogenic	rs267607277
16-56370693-G-A	SUDCRRC-096	De novo	*GNAO1*	c.644G > A	p.Cys215Tyr	NA	NA
1-237608788-C-T	SUDCRRC-046	De novo	*RYR2*	c.1258C > T	p.Arg420Trp	Pathogenic	rs190140598
1-237798237-C-T	SUDCRRC-100	De novo	*RYR2*	c.6737C > T	p.Ser2246Leu	Pathogenic	rs121918597
2-166929950-A-G	SUDCRRC-010	Mosaic	*SCN1A*	c.182T > C	p.Leu61Pro	NA	NA
19-55663261-G-A	SUDCRRC-037	De novo	*TNNI3*	c.574C > T	p.Arg192Cys	Likely_pathogenic	rs727503499

The variant ID provided (Var_ID_GRCh37) consists of the variant chromosome, position reference and alternate allele on the human reference genome build 37. We have provided corresponding variant annotation information for the HGVS coding sequence (HGVS_c) and the HGVS amino acid sequence (HGVS_p). We have also provided two columns that describe whether or not at time of analysis the listed variant in question had a clinical significance in ClinVar defined as pathogenic or likely pathogenic (ClinVar_ClinSig) and a corresponding column for the corresponding rsID for easier lookup in ClinVar. A variant that was absent from ClinVar at time of analysis has ClinVar_ClinSig and ClinVar_rsID entries of “NA.” All variants within this table (including the ones not previously reported in ClinVar) were classified as pathogenic or likely pathogenic by our team of geneticists and clinicians involved in this study.

### Burden of Loss-of-Function DNMs in SUDC Probands.

We tested the burden of loss-of-function (LoF) DNMs in SUDC probands relative to expectation based on mutation rate and burden within external control trios to determine if there was an excess of these variants, particularly within LoF-intolerant genes. LoF variants are required to have stop-gain, frameshift, or splice donor/acceptor annotation in protein coding genes. We define genes as LoF-intolerant if they have an LoF observed/expected upper-bound fraction (LOEUF) < 0.35 in gnomAD v2.1 (*n* = 3,219) (24, 25). We also defined a final set of genes that have a gnomAD v2.1 LOEUF < 0.35 along with at least one observation of an LoF DNM in denovo-db v1.6.1, called in a sample with a defined noncontrol phenotype (*n* = 505, denovo-db) (26).

We found evidence for excess de novo LoF variants in genes impacted with similar variants in individuals with developmental disorders. There was insufficient evidence for an excess of LoF DNMs across the exome or in LoF-intolerant genes as defined above (Tables 2 and 3). Although our trio cohort size was underpowered for exome-wide analysis, we found a signal within LoF-intolerant genes hit with LoF DNMs in denovo-db (26) compared to expected rate (observed = 3, expected = 0.7, *P* = 0.03) (Table 2) and to external control trios (odds ratio = 6.73, *P* = 0.02) (Table 3). The three impacted genes comprised *MYO9B*, an unconventional myosin that interacts with actin and RhoA; *RASGEF1A* (a GTPase preferentially expressed in brain); and *LMTK3* (tyrosine kinase expressed selectively in brain) (27, 28). None of these genes have been implicated in sudden death. Their pattern of expression is consistent with involvement of both heart and brain in SUDC pathology.

**Table 2.** Comparison of de novo LoF mutation burden in our SUDC cohort relative to expected mutation rate

Geneset	Observed	Expected	Enrichment	*P* value
Exome	15.00	10.40	1.45	0.10
LoF-intol	5.00	3.12	1.60	0.21
LoF-intol and ≥1 LoF in denovo-db	3.00	0.70	4.07	0.03

Comparison of de novo LoF mutation burden in our SUDC cohort relative to expected mutation rate. The comparison reaches nominal significance when the comparison is narrowed to genes that are both LoF-intolerant have at least one LoF de novo mutation in noncontrol trios within denovo-db (26).

**Table 3.** Comparison of de novo LoF mutation burden in our SUDC cohort relative to healthy control trios

Geneset	Odds ratio	95% CI lower	95% CI upper	*P* value
Exome	1.52	0.81	2.71	0.14
LoF-intol	2.13	0.64	5.57	0.11
LoF-intol and ≥1 LoF in denovo-db	6.73	1.11	29.9	0.02

Comparison of de novo LoF mutation burden in our SUDC cohort relative to healthy control trios. The comparison reaches nominal significance when the comparison is narrowed to genes that are both LoF-intolerant have at least one LoF DNM in noncontrol trios within denovo-db (26).

These findings led us to consider related questions about the gene variants in our trio analysis, seeking to evaluate the deleteriousness of these variants and to explore potential interactions among gene products.

### Analysis of Combined Annotation-Dependent Depletion and Protein–Protein Interaction Networks.

**Fig. 3.**
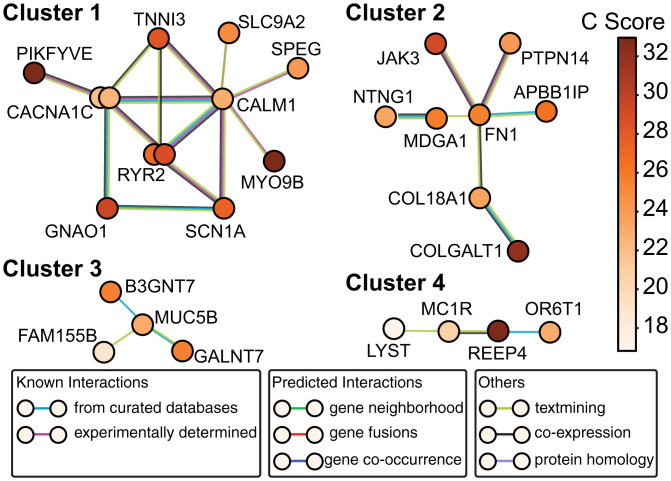
Protein–protein association network analysis of SUDC-associated de novo and transmitted mosaic nonsynonymous mutations. A total of 79 proteins were analyzed for PPIs using STRING v11 (29). The cutoff for consideration was mutational deleteriousness marked by a CADD score > 15 [the suggested and conventional cutoff (30)]. A total of 32 edges (functional associations) were observed vs. 23 expected (*P* = 0.036), suggesting that the SUDC-associated proteins are concentrated in certain pathways. In contrast, the 49 proteins with CADD score < 15 (including 29 synonymous mutations and 20 missense mutations) formed 2 connections vs. 4 expected (*P* = 0.923). For clarity, only clusters with more than two proteins are shown. Cluster 1 consists of genes almost entirely represented in the CardiacEpilepsy geneset, with *CACNA1C* and *RYR2* each represented by two mutations from different probands. Cluster 2 reflects PPIs among gene products involved in neural development. Cluster 3 includes genes encoding protein glycosylating enzymes. Cluster 4 includes genes for proteins involved in cytosolic organelle trafficking. The circles of corresponding genes are color-coded according to their CADD (*C*) scores, with darker shades representing more deleterious mutations predicted by CADD. There are two DNMs each from *CACNA1C* and *RYR2*, respectively (represented by the double circles). Edges are color-coded according to different degrees of interaction evidence as explained by the legend from STRING. The level of evidence goes from high (curated databases/experimentally determined) to low (text mining, where gene symbol cooccurrences in the same sentence/paragraph/document were considered with different weights).

To look in an unbiased way for structural or functional groupings of products of genes hit by nonsynonymous DNMs in SUDC, we analyzed protein–protein interactions (PPI) with STRING (29). STRING enables use of publicly available protein–protein association data to determine if an input set of genes code for proteins that are enriched for interactions. Here we chose to analyze functional interactions in order to capture more gene networks. In STRING, each gene symbol is represented as a node, and each PPI is represented as an edge. Edges are further categorized based on the level of evidence as shown in Fig. 3. No dyadic PPIs (edges) were found between genes with synonymous variants in the SUDC dataset (*n* = 30, *P* = 1). In contrast, 48 edges were observed among the genes with nonsynonymous variants (*n* = 98), in large excess of the 29 predicted (*P* = 0.00072).

We also categorized DNMs by their deleteriousness (30), as quantified by the combined annotation-dependent depletion (CADD) score (see Fig. 3 legend for details). We used a conventional cutoff (CADD score > 15; variant in the top ∼3% in terms of deleteriousness of all possible variants). This screened out all but one of the synonymous mutations and an additional 20 missense mutations, leaving a majority (*n* = 79) of the initial set of burdened genes for further consideration. Variants with the CADD score > 15 (*n* = 79) displayed 32 edges vs. 23 expected (*P* = 0.0358). In contrast, variants with CADD score < 15 (*n* = 49) displayed only two edges relative to four expected (*P* = 0.923), the expectation allowing for the proclivity of specific genes to interact. This analysis indicates that the more deleterious DNMs in SUDC probands tend to occur within known protein networks.

In a graphic representation of PPI-based gene (product) clusters (Fig. 3), the largest cluster incorporated *CACNA1C* (×2), *RYR2* (×2), *CALM1*, *TNNI3*, *GNAO1*, and *SCN1A*: every one of the CardiacEpilepsy genes implicated in DNM + transmitted mosaic analysis (Table 1). *PIKFYVE* and *MYO9B* (both impacted with LoF DNMs) were also members of the same highly interconnected PPI network, tying specific LoF variants back to the original CardiacEpilepsy set. Cluster 1 represents an unbiased replication of the manually curated CardiacEpilepsy geneset, and offers support for a core group of risk genes for sudden death that are enriched for highly disruptive genetic variants in SUDC cases.

The PPI analysis (29) also uncovered additional clusters of genes beyond cluster 1 (Fig. 3), with smaller cluster sizes and fewer intracluster interactions. Although no direct evidence supports their role in SUDC, they might suggest potential pathogenicity beyond CardiacEpilepsy genes. Specifically, cluster 2 includes genes involved in neural development, such as fibronectin1 (*FN1*) and netrin G1 (*NTNG1*). Cluster 3 incorporates genes encoding protein glycosylating enzymes *GALNT7* and *B3GNT7*. Cluster 4 includes genes for proteins involved in cytosolic organelle trafficking (LYST, REEP4). To rule out the possibility that genes from clusters 2 to 4 are identified in our DNM analysis simply due to their large size and thus greater proclivity for mutations, we analyzed the coding sequence sizes of genes in all clusters. The average coding length in PPI clusters 2 to 4 was ∼5 kB, roughly half that in cluster 1 (*SI Appendix*, Fig. S2); this excludes greater coding length as a simple explanation for picking up these gene variants. In summary, our PPI analysis objectively identified the gene network involved in CardiacEpilepsy along with additional smaller networks that might also play a role in SUDC.

### Potential Overlap of SUDC-Related Genes with Gene Sets Associated with Other Disorders.

Given that our PPI analysis (29) cross-referenced members of clusters 2, 3, and 4 to other disorders, we asked if SUDC-related genes significantly overlap with disorders outside the CardiacEpilepsy set. We therefore widened our analysis to neurodevelopmental disorders not associated with death (e.g., autism spectrum disorders [ASD] and developmental disorder [DD]). From the SUDC gene set, those genes affected by a mutation with a CADD score > 15 (*n* = 79) appeared more frequently than chance in gene lists for ASD (*P* = 1.67 × 10^−3^) and for DD (*P* = 3.03 × 10^−3^, binomial test) (Dataset S6). Specifically, a gene set for ASD (31) includes three genes in cluster 1 of the STRING analysis (*CACNA1C*, *MYO9B*, *SCN1A*). A geneset for DD (32–34) incorporates four genes in cluster 1 (*CACNA1C*, *GNAO1*, *SPEG*, *SCN1A*), four genes in cluster 2 (*FN1*, *COL18A1*, *JAK3*, *PTPN14*), and another gene in cluster 4 (*LYST*) (Dataset S7). In contrast, no significant enrichment of members of the SUDC gene list were found in gene sets for asthma, ACMG rare diseases absent cardiac phenotypes, hereditary pediatric cancers (Invitae), or DNM in pediatric cancer (MuSIC) (Dataset S6). Overall, this analysis suggests that SUDC genetic architecture may have some overlap with that of neurodevelopmental disorders.

### Ca^2+^ Regulation in Cardiomyocyte and Neuronal Excitability.

**Fig. 4.**
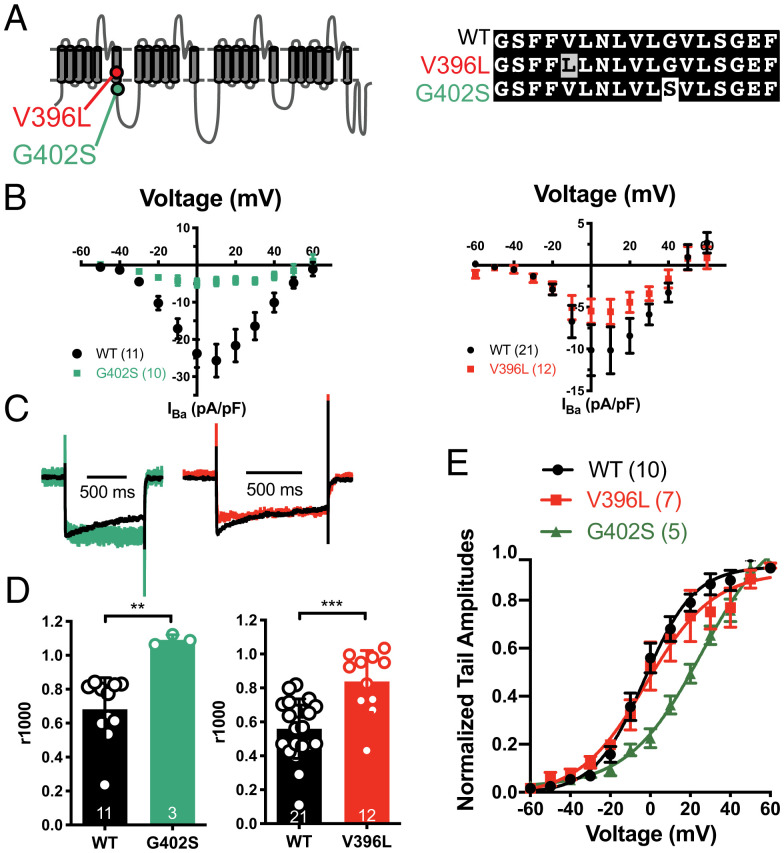
SUDC DNMs G402S and V396L of the *CACNA1C* gene lead to decreased Ba^2+^ current density and slowed inactivation. (*A*) A schematic diagram showing the positions of the two SUDC DNMs in *CACNA1C*. Gray highlight connotes a subtle change, white highlight, a more drastic alteration. (*B*) I-V curves showing the overall activation profile with 30 mM Ba^2+^ as charge carrier. (*C*) Averaged traces from wild-type, G402S, and V396L cells were superimposed to show the differences in inactivation. (*D*) Quantification of the ratio between the residual current at 1,000 ms and the peak current (r1000). **P < 0.01, ***P < 0.001 by unpaired two-tailed Student’s *t* test. (*E*) Activation curves for WT and V396L obtained by Boltzmann fit to normalized tail current amplitudes (*V*_h_ = -2.9 mV and -2.2 mV, *k* = 12.71 and 16.86, respectively).

Six of eight mutations in cluster 1 affected genes involved in Ca^2+^ regulation, including two cardio-neuro excitability genes having nonsynonymous DNMs in two cases each. Two cases had pathogenic variants in *RYR2* (MIM# 180902) (35, 36), encoding the ryanodine receptor 2 (RYR2) protein and previously studied in mouse models (37, 38). Based on expected mutation rate, the probability of observing ≥2 de novo missense pathogenic variants in *RYR2* among 124 cases is *P* = 1.7 × 10^−7^. *RYR2* gain-of-function variants cause catecholaminergic polymorphic ventricular tachycardia 1, producing lethal cardiac arrhythmias. Two cases had Sanger-validated variants in *CACNA1C* (MIM# 114205), which encodes a voltage-dependent L-type calcium channel pore-forming subunit (known as α_1C_ or Ca_V_1.2). Both *CACNA1C* variants were in a 104-nucleotide exon (exon 8, chr12:2613601–2613705) that strongly influences channel opening and closing and is critical in cardiac function (Fig. 4*A*). Exon 8 and its mutually exclusive splicing counterpart, exon 8A, harbor pathogenic mutations in Timothy syndrome, characterized by developmental delays, neuropsychiatric comorbidities, and long QT syndrome (39, 40). The probability of observing ≥2 mutations across our 124 trios specifically within exon 8 is *P* = 1.0 × 10^−7^. Largely because of these genes, the overall SUDC gene set showed significant overrepresentation of genes implicated in β-adrenergic signaling (*CACNA1C*, *RYR2*, *VAMP8*) by PANTHER overrepresentation test (enrichment factor 14.25, *P* = 2.38E-04).

Another DNM was found in *CALM1*, encoding the archetypical Ca^2+^ sensor calmodulin, mutations of which have been found in cardiac arrhythmias (41, 42). The identical mutation, p.Asn98Ser, was reported in a 4-y-old girl with sudden cardiac death from sinus bradycardia and stress-induced bidirectional ventricular ectopy (43). A DNM was also found in *TNNI3*, the inhibitory subunit of troponin in cardiac muscle, part of the thin-filament regulatory complex that confers calcium-sensitivity to myocardial contraction. Dysfunction of contractile machinery can be arrhythmogenic via Ca^2+^ handling (44). Indeed, our *TNNI3* case demonstrated moderate myocyte hypertrophy, myocyte disarray in the ventricular septum, and increased heart weight. Taken together, the six cases with mutations—*RYR2* (×2), *CACNA1C* (×2), *CALM1*, and *TNNI3*—suggest that Ca^2+^ dysregulation may play an important role in SUDC.

### Functional Characterization of G402S and V396L CACNA1C Mutations.

To more fully characterize the electrophysiological consequences of the two mutants in L-type Ca^2+^ channels, we compared the *CACNA1C* G402S and V396L mutations with wild-type channels in HEK293T cells using whole-cell patch clamp recordings. The channels’ current–voltage relationship curves, expressed as peak current normalized by cell membrane area (pA/pF), showed reduced Ba^2+^ current density for both G402S and V396L (Fig. 4*B*). In recordings with robust Ba^2+^ currents, voltage-dependent inactivation was nearly abolished for both mutations (Fig. 4 *C* and *D*), consistent with previous reports in G402S (39, 45). We measured tail currents following brief depolarizing pulses to identify possible differences in the voltage-dependence of channel activation. Similar to previous observation by Dick et al. (45), we saw a significant rightward shift of G402S voltage-activation curve comparing to wild-type. In contrast, the activation curve of V396L is indistinguishable from that of wild-type (Fig. 4*E*). This suggests that changes in inactivation are not solely dependent on voltage shifts in activation. Our experiments indicate that attenuation of L-type channel inactivation is a common denominator of SUDC-associated *CACNA1C* and *CALM1* mutations.

### Overtransmission of Deleterious Variants in Cardiac/Epilepsy Genes from Heterozygous Parents.

We tested for overtransmission of heterozygous deleterious variants present in parents to probands involving cardiac or seizure genes with an excess of DNMs in our cohort. We defined deleterious variants as: 1) LoF in a gene with LOEUF < 0.35 or 2) missense pathogenic or likely pathogenic in ClinVar (46). A total of 14 variants were found across the 248 trio parents that met these criteria. The null expectation (where these variants have no role in any of the 124 sudden death cases) is that ≤50% of these variants (i.e., ≤7) would be transmitted. We instead observed excess variant transmission (11 of 14 variants transmitted, fraction = 0.79, one-sided binomial *P* = 0.03). This suggests that some nonzero fraction of these transmitted variants may be a part of the genetic risk profile of the recipient probands. Variant transmission test statistics should not be affected by parent ancestry, and consistent with this, the results of this test were similar when performed on explicitly European-ancestry parents (9 of 12 variants transmitted, fraction = 0.75, *P* = 0.07). A similar test for overformation of recessive genotypes across the exome was underpowered (one of two genotypes formed, fraction = 0.5, one-sided binomial *P* = 0.4375).

Among variants transmitted from heterozygous carrier parents within cardiac or seizure disorder genes, two variants associated with autosomal dominant conditions were classified as contributory to death in two cases (*SCN1A* and *DEPDC5*) (Datasets S2 and S8). We also identified biallelic variants in *PPA2* in a proband (Datasets S2 and S9) that were implicated in sudden cardiac death and contraction bands evident in the cardiac muscle fibers (47–49). This compound heterozygous genotype was also observed in the proband’s sibling whose death was originally classified as SIDS (49). Combining these numbers with the total transmitted parental-mosaic variants classified as pathogenic, we found that a total of 3 of 124 trios (2.4% of the cohort) carried a genotype that was not de novo or parental-mosaic in origin but likely contributed to sudden death. Within the callsets described in this study, we thus found variants that were likely contributory to death in a total of 11 of 124 cases, a yield of around 8.9%.

## Discussion

Our trio-cohort whole-exome sequencing study of SUDC identified a highly significant, nearly 10-fold, enrichment of nonsynonymous mutations in cardio-neuroexcitability genes compared to nonsudden death controls. Pathogenic variants in cardio-neuroexcitability genes were found in 9% of cases. Most of these pathogenic DNMs altered calcium-related regulation of cardiomyocyte and neuronal excitability at a submembrane junction, implicating this as a key pathway in sudden pediatric deaths. Our functional studies revealed attenuated L-type channel inactivation in our SUDC-derived *CACNA1C* mutations. Furthermore, LoF or missense pathogenic variants in cardiac and seizure disorder genes were significantly overtransmitted to decedents. Finally, our analysis revealed a trend for excess LoF mutations in potentially disease-relevant genes that are intolerant to LoF variants, supporting novel genetic causes of pediatric sudden deaths that could be discovered with larger cohorts.

### Limitations of the Current Study.

In the most definitive population-based survey, the CDC provides multiyear, nation-wide epidemiological statistics and systematically distinguishes between explained vs. ill-defined or undetermined causes of fatality (5). One limitation of our study is that the case ascertainment was not population-based, since it arose from referrals from bereaved parents, the child’s clinician, or the medical examiner or coroner who initially investigated the death. This might explain why the proportion of children 1- to 4-y-old in this study (110 of 124 or 88.7%) was not in complete agreement with that reported by the CDC (203 of 342 or 59% in 2019). We also focused exclusively on unexplained deaths, which might explain why distributions of age in our cohort are not strictly comparable to recent population-based studies of sudden death in infancy and childhood, wherein a large majority of cases were not unexplained, but classified as definite or possible cardiac or SUDEP (50).

Our DNM calling pipeline relied heavily on hard call QC thresholds, and may have missed real mutations that are contributors to sudden death. For example, the requirement for a variant to be found in at least 30% of reads excluded a de novo frameshift mutation in *DNMT3A*, found in ∼28% of reads and isolated in a secondary analysis of the same sudden death cases. The mutation in this developmental delay/autism risk gene might exemplify LoF DNMs within genes that are LoF-intolerant.

Our genetic study on a relatively rare and unusual cohort faces inherent limitations for future replication, but we note recent studies on similar cohorts (51) that have identified similar genes and variants, such as a *CACNA1C* LoF variant. Finally, the mosaicism analysis of our cohort has been primarily limited to DNA from blood; future studies that draw from multiple organ DNAs will yield more insights into the nature of the disease.

### Sleep as Predominant Behavioral State at Death of SUDC Decedents.

Previously healthy athletes, adolescents, and young adults who die suddenly typically do so while awake and active or exercising (52, 53). In contrast, most SUDC cases (75 of 79) with DNMs were asleep or resting at the time of death (Dataset S2), including 50% of those with DNMs affecting genes involved with Ca^2+^ physiology in heart and brain (*CACNA1C*, *RYR2*, *CALM1*, *TNNI3*). Among these six patients, three were physically active or in emotionally stimulating settings, two were asleep and one was in bed but with uncertainty if quietly playing or asleep. Because this gene set is overrepresented with genes implicated in β-adrenergic signaling, it is conceivable that increased sympathetic tone may predispose some sleeping toddlers to cardiac arrhythmias. The patient with the *GNAO1* mutation was asleep. One of two carriers of pathogenic *SCN1A* variants in this study, along with three other SUDC cases with *SCN1A* pathogenic variants in our registry but outside of this study, were all sleeping at the time of death, typical of SUDEP cases (7).

### The Relationship between SUDC and SUDEP.

We hypothesized that epilepsy genes may contribute to death in some SUDC cases due to the high frequency of febrile seizures in SUDC cohorts (10–12). Although multiple genes have been associated with febrile seizures (54), it is likely that its full genetic architecture has not yet been fully characterized. Genes such as *SCN1A* have variable expressivity and reduced penetrance. A spectrum of diverse phenotypes exists, from severe epileptic encephalopathies to only febrile seizures to asymptomatic (55). DNMs are common among the severe Mendelian epilepsy syndromes (56). The proband with the inherited parental-mosaic *SCN1A* variant (case 10), for example, likely died from a febrile seizure. If she had lived longer or her mutation was identified later in life, she would probably be diagnosed with Dravet syndrome and her death an SUDEP. This case highlights a continuum between some febrile seizure-associated deaths and SUDEP. Pathogenic variants in epilepsy and cardiac disorder genes occur in some SUDEPs, supporting parallel mechanisms in sudden deaths associated with febrile and afebrile seizures (1). The elevated rate of febrile seizures in sudden explained childhood deaths suggests that in some cases, the “explained” cause of death was inaccurate, and their febrile seizures were lethal (4, 10).

### Role of Calcium Regulation-Related Genes in SUDC.

Six of eight pathogenic variants identified as likely causes of death (Table 1) disrupt the flow of calcium ions in cardiac myocytes during heart excitation directly or indirectly: *RYR2* (controls calcium flow out of sarcoplasmic reticulum), *CACNA1C* (controls calcium flow into myocytes), and *CALM1* (mediates calcium-dependent regulation of channels encoded by *CACNA1C* and *RYR2*). We found the *TNNI3* variant to be associated with myofibrillary derangement (Table 1), which is often found to indirectly alter calcium homeostasis and engender cardiac arrhythmias (44). Both *RYR2* R420W and S2246L are linked to catecholaminergic polymorphic ventricular tachycardia (CPVT) (35, 36); *CALM1* N98S is linked to long QT syndrome, CPVT, idiopathic ventricular fibrillation, and sudden unexplained death (41). Mouse models have been made to study the mechanisms of these three mutations. All three available knockin mouse models showed Ca^2+^ dysregulation, often with physiological phenotypes mimicking each other: in *Ryr2*^R420W/R420W^ mice, the amplitude of the depolarization-induced Ca^2+^ transient was significantly reduced while the decay time from the peak was prolonged (37); in *CALM1*^N98S/+^ knockin mice, the degree to which β-adrenergic stimulation increased local Ca^2+^ and slowed inactivation exceeded that in wild-type littermates (57); in both *CALM1*^N98S/+^ and *Ryr2*^S2246L/+^ mice, epinephrine-induced ventricular tachycardia was observed (38, 57).

The distinct electrophysiological properties of the two *CACNA1C* mutations shed light on L-type Ca^2+^ channelopathies. Two *CACNA1C* mutations causing Timothy syndrome (G402S and G406R) displayed slowed inactivation but with opposite effects on channel activation (45). We saw similar effects of G402S, and further found that V396L, a third mutation in the same region, also slows inactivation but with preserved channel activation (Fig. 4*E*). Of note, the *CACNA1C* G402S variant was first reported by Splawski et al. (39) as a case of type 2 Timothy syndrome (TS2), wherein the patient developed normally but had a nonfatal cardiac arrest at age 4. Since then, at least three similar cases with the same G402S variant have been reported, all of whom showed normal development but suffered cardiac arrests at various ages (2 mo to 13 y) (58, 59). This highlights the impact of G402S on cardiac function rather than on other aspects seen in type 1 Timothy syndrome (TS1), such as immune system dysfunction and syndactyly (37).

These calcium-related genes are also expressed in neurons and, as with primarily neural genes (e.g., *SCN1A*), dual cardiac-brain dysfunction may contribute to sudden death. The encoded proteins work in close proximity, near a special intracellular space, the dyadic cleft in cardiomyocytes or endoplasmic reticulum–plasma membrane junction in neurons (*SI Appendix*, Fig. S3). In both cell types, ionic pathways sensitive to a rise in local submembrane Ca^2+^ can generate a pathogenic delayed afterdepolarization and elevation of excitability (44, 60, 61). The local Ca^2+^ rise may activate sodium entry via the sodium-calcium exchanger (62–64) or nonselective cation channels (60, 62, 65). Thus, the pathophysiological mechanism in heart and brain may arise from abnormal local Ca^2+^ signaling and its specific consequences on tissue-level electrophysiology.

### The Continuum of “Cardiac” and “Brain” Genes and Their Role in SUDC.

Our study supports that some cardiac and brain genes form an overlapping continuum rather than discrete categories. Many ion channel and calcium regulatory genes are expressed in heart and brain. Patients with cardiac gene mutations have increased rates of neurological disorders: high rates of epilepsy in patients with LQT2 syndrome due to *KCNH2* mutations (66) and high rates of neurodevelopmental delay in patients with CPVT due to RYR2 mutations (67). Conversely, induced pluripotent stem cell–derived cardiomyocytes from patients with Dravet syndrome due to SCN1A mutations have abnormal contractility (68). Future research to explore the functional consequences in both heart and brain, and their interactions, may advance our understanding of pathophysiology and identify preventive and therapeutic strategies.

Our present results suggest that Ca^2+^ intracellular regulation may be a pathway frequently perturbed in SUDC cases. Sudden death in children likely results from diverse death-promoting genetic and environmental factors. Some genetic risk factors may act in a pleiotropic manner, increasing risk for multiple phenotypes at once. A larger sample may reveal other key pathways perturbed in SUDC cases. The present data indicate that deleterious DNMs are significant genetic risk factors for childhood sudden unexplained death, and that their identification may lead to medical intervention that ultimately saves lives.

## Materials and Methods

### Sample Recruitment.

We performed detailed phenotyping and trio whole-exome sequencing in 129 consecutively enrolled decedents in the New York University (NYU) Sudden Unexplained Death in Childhood Registry and Research Collaborative (ClinicalTrials.gov Identifier: NCT03109197). Cases were referred by our network of medical examiners, coroners, and clinical care physicians or parents self-referred and, therefore, our cohort is not population based. Parental consent was obtained in this NYU Institutional Review Board approved study (# S14-01061). DNA was extracted from parent blood samples and child decedent blood (or postmortem tissue samples when blood was unavailable).

### Whole-Exome Sequencing and Data Processing.

Exomes (127 of 129 trios) were sequenced at Columbia University Institute for Genomic Medicine. High-quality sequence data, alignment metrics, and pairwise nonrelatedness patterns were present in 124 of 129 trios that were analyzed. Methodology for sequencing, data alignment, and variant calling is summarized in *SI Appendix*, *Supplementary Methods*. Genetic data analyzed by M.H., with interpretation by O.D., M.J.A., D.J.T., R.R., and J.G.P.

### Coverage Statistics in Sequence Data.

Coverage across consensus coding bases in SUDC probands was more than sufficient for variant calling. The average overall coverage across these bases in probands was 89×; only two probands in the full cohort had <50× coverage. We found that on average, 97.4% of consensus coding bases were covered at least 10×, and that in addition an average of 95.1% of these bases were covered at least 20×. These statistics suggest that if a variant was present in a proband coding base, we were well powered to detect it.

### Control Cohorts.

Each variant call was absent in 9,739 unrelated internal controls negative for cardiac, pulmonary, and neurological disorders, sequenced on the same exome kits.

We used two separate trio cohorts for case/control comparisons: 1) for an internal control, 574 internal trios with obsessive-compulsive disorder (not associated with sudden death); 2) for an external control, 1,911 healthy controls sequenced elsewhere (19).

### DNM Calling and Screening.

Candidate DNMs were identified in our 124 trios and 574 internal “controls” trios using the ATAV “list-trio” function with conservative thresholds of coverage and quality control (*SI Appendix*, *Supplementary Methods*). We rejected DNMs whose underlying sequence alignment data failed visual inspection. We excluded trios with ≥6 DNMs. We were left with 124 SUDC cases and 573 internal controls.

### Parental Mosaic Genotype Calling.

We called transmitted high-confidence parental mosaic variants in cases and controls using ATAV’s “list-parental-mosaic” function (*SI Appendix*, *Supplementary Methods*) (69).

### Characterizing Deleteriousness of Mutations.

In addition to classifying mutations as synonymous/nonsynonymous, we also used CADD to generate a more stringent measure of mutation deleteriousness. In the CADD system, a support vector machine is trained based on a wide range of data types to predict the deleteriousness of a given mutation. The resulting CADD score is a scaled transformation of the deleterious ranking of the mutation among all possible 8.6 billion substitutions in the human genome. The scaled score is calculated as −10 × log_10_(ranking), thus top 10% in the ranking is assigned with a CADD score of 10, top 1% in the ranking with a CADD score of 20, and 0.1% with a CADD score of 30, and so on.

### Electrophysiological Characterization of CACNA1C G402S and V396L DNMs.

To identify possible differences in electrophysiological properties, we compared the CACNA1C G402S and V396L mutations with wild-type in HEK293T cells using whole-cell patch clamp recordings. Ba^2+^ currents were recorded in HEK293T cells expressing Ca_V_1.2 wild-type, G402S, or V396L cDNAs, together with β_2a_ and α_2_δ_1_ cDNAs 48 to 60 h after transfection (*SI Appendix*, *Supplementary Methods*). Electrophysiological analysis was done by R.W.T., X.W., and G.G.

## Supplementary Material

Supplementary File

Supplementary File

Supplementary File

Supplementary File

Supplementary File

Supplementary File

Supplementary File

Supplementary File

Supplementary File

Supplementary File

## Data Availability

Whole-exome sequencing data from this study can be requested through the study authors. At the time of publication, the authors are in the process of obtaining IRB approvals to deposit available data into a publicly accessible database. All other study data are included in the article and *SI Appendix*.
