# Supplementary material for: De novo mutations in childhood cases of sudden unexplained death that disrupt intracellular Ca2+ regulation
Source: Proc Natl Acad Sci U S A. 2021 Dec 20;118(52):e2115140118. doi: 10.1073/pnas.2115140118 (PMC8719874; doi:10.1073/pnas.2115140118)
Supplement: Supplementary File [file pnas.2115140118.sapp.pdf]

PNAS
www.pnas.org

Supplementary Information for

De novo mutations in childhood cases of sudden unexplained death that disrupt intracellular Ca^{2+} regulation

Matthew Halvorsen, Laura Gould, Xiaohan Wang, Gariel Grant, Raquel Moya, Rachel Rabin, Michael J. Ackerman, David J. Tester, Peter T Lin, John G. Pappas, Matthew T. Maurano, David B. Goldstein, Richard W. Tsien, Orrin Devinsky

Corresponding authors: Orrin Devinsky and Richard W. Tsien

Email: od4@nyu.edu (O.D.) or richard.tsien@nyulangone.org (R.W.T.)

This PDF file includes:

- Supplementary text
- Figures S1 to S3
- Index of Datasets S1 to S9
- SI References

Other supplementary materials for this manuscript include the following:

- Datasets S1 to S9

Supplementary Information Text

SUDCRRC Study Group Investigators (Alphabetically):

T Andrew, MD[6], JC Downs, MD[7], D Friedman, MD[2], H Jarrell, MD[8], K Landi,MD[2], K Lear, MD[9], J Lee, BS[2] ,K Maloney, MD[10,11], JJ Maleszewski[4], K Pinckard, MD[12], K Pinneri, MD[13], R Reichard, MD[4], K Roman, MD[2],A Williamson, MD[14]
2 NYU Langone, New York, NY; 4 Mayo Clinic, Rochester, MN; 5 Columbia University, New York, NY; 6 White Mountain Forensic Consulting Services, Concord, NH, 7 Charleston Southern University, Charleston, SC; 8 University of New Mexico, Albuquerque, NM; 9 Arapahoe County Coroner's Office (CO), Centennial, CO; 10 Erie County Medical Examiner's Office, Buffalo, NY; 11 University at Buffalo School of Medicine, Buffalo, NY; 12 Travis County Medical Examiner, Austin, TX; 13 Montgomery County Forensic Services Department, Conroe, TX; 14 Zucker School of Medicine at Hofstra / Northwell, Hempstead, NY

Supplementary Methods

Storage and retrieval of variant calls

All sample-level variant calls as well as the parameters underlying each call were stored in a relational database at IGM known as annoDB. Retrieval and thresholding of these calls, as well as the inference of novel genotypes in family-based data using single sample calls, was done using ATAV v7.0.17.

De Novo mutation calling and screening

We pulled down a set of candidate *de novo* mutation calls using ATAV's 'list-trio' function. For the initial set of candidate *de novo* mutation calls, the following QC thresholds were applied: coverage >= 10x; >= 3 reads supporting call; SNV VQSR tranche < 99%; indel QD>=2; indel FS < 200; indel ReadPosRankSum > -20; QUAL >= 30; QD >= 1; GQ >= 20; no status as an artifact based on internal records or EVS 'FAIL' assignment; coding region annotation in at least one CCDSr20 transcript; MAF < 0.01 in internal controls, as well as gnomAD global and subpopulation cohorts. To extract a set of high confidence *de novo* mutation calls from this lower confidence callset, we further more required that calls meet the following criteria: variant classification of 'DE NOVO' via ATAV 'list-trio' function; MAF < 0.0001 in internal controls and gnomAD Exome plus Genome global and subpopulations; no internal controls that have a QC fail for the variant call based on QC parameters for initial list-trio function call; status of 'pass' for variant call in proband; at least 30% of reads in child supporting variant call; >=10x coverage in proband and each parent; MQ>=40, QD>=2 and QUAL>=30 in proband. We additionally filtered out any call found in greater than 5% of parent reads at the call site. Finally, we required that each included proband had no more than 5 *de novo* mutation calls that met these criteria. Any probands that had a mutation count that exceeded this threshold were excluded from DNM analysis. All SUDC case *de novo* mutations were visually inspected in IGV to make sure the underlying alignment was sound. Any variant call that failed visual inspection was excluded from the final callset.

Transmitted parental mosaic genotype calling

We called transmitted high-confidence parental mosaic variants in all trio cases and controls using ATAV's 'list-parental-mosaic' function. In doing so we applied the following QC thresholds: MAF <0.0005 in global and ethnic subpopulations of internal controls and gnomAD; SNV VQSR tranche < 99%; indel QD>=2; indel FS < 200; indel ReadPosRankSum > -20; QUAL >= 30; MQ>=40; RPRS>-4; MQRS>-8; child QD>=2; child binomial P > 0.001; parent binomial P < $5x10^{-6}$; child percent alt read between 30 and 80%; no status as an artifact based on internal records or EVS 'FAIL' assignment; annotated as affecting a coding region in at least one CCDSr20 transcript. The genotype calls from this screen were considered our final parental mosaic genotype callset. We note that here, the MAF threshold was raised slightly compared to the threshold of 0.0001 used in de novo mutation calling, since 1) these calls are exceedingly rare, and 2) it stood to reason that some could be incompletely penetrant, particularly if the variants were found in a substantial fraction of parental cells.

Transmitted parental heterozygous genotype calling

We called all variants across individual samples that reasonable call QC. The thresholds used include: MAF < 0.005 in internal controls and gnomAD Exome plus Genome global and subpopulations; >=10x coverage; MQ>=40; QD>=2; QUAL>=30; GQ >= 20; exclusion of variants that have been derived as artifacts based on internal sequence data; SNV VQSR tranche < 99%; no EVS classification of 'FAIL'. A higher MAF threshold was used here than in extraction of de novo and transmitted parental-mosaic variant calls to capture pathogenic genotypes that followed both dominant and recessive (where MAFs will be slightly higher) patterns of inheritance. We kept the subset of sample-level calls that were either loss of function (stop-gained, frameshift, or splice

donor/acceptor annotation) and within a gene with gnomAD v2.1 loss-of-function observed/expected upper bound fraction <0.35, or missense and annotated as 'Pathogenic' or 'Likely Pathogenic' in ClinVar version 2019-02-19. We pulled down all variants that met these criteria across SUDC parents in cardiac or epilepsy genesets described, and then identified the subset of these calls that were also found in SUDC probands. We tested for overtransmission of these variants to probands via a one-sided binomial test looking at the percentage of parental variants transmitted to SUDC probands, with the null hypothesis that the portion of variants transmitted will be less than or equal to 50% of all variants included across the cohort.

Definition of Ancestry

Ancestry was defined based on the genetic data, rather than that which was reported for each sample. For each sample, a probability of membership to each of six possible ancestries was computed. For further details, see (1). Here, any tests focused explicitly on European-ancestry individuals required a 'Caucasian_prob' value > 0.95.

Electrophysiology

HEK293T cells in 35 mm dishes were transfected with 2 μg rat $Ca_V1.2$ WT or G402S or V396L pcDNA plasmids together with 1 μg β2a, 1 μg α2δ1 and 0.05 μg mCherry pcDNAs. Cells were split on 12 mm #1 coverslips around 36 hours after transfection, and whole cell recording was performed at room temperature 48-60 hours after transfection. Data were collected through MultiClamp 700B Microelectrode Amplifier and pCLAMP11 software (Molecular Devices, CA). Pipette resistance was 4-8 MΩ when loaded with the intracellular solution and immersed in the extracellular solution. Series resistance and membrane capacitance were compensated up to 80%. The intracellular solution contained (in mM): 132 CsCl, 10 Tetraethylammonium chloride (TEA-Cl), 10 EGTA, 1 $MgCl_2$, 3 Mg-ATP, 5 HEPES, pH 7.3 adjusted by CsOH. The external solution contained (in mM): 79 NaCl, 20 TEA-Cl, 30 $BaCl_2$, 5 CsCl, 1 $MgCl_2$, 10 HEPES, 10 glucose, pH 7.3 adjusted by NaOH. The osmolarity is 295 mOsm for the intracellular solution, and 305 mOsm for the extracellular solution. The membrane voltage was depolarized in 1-second steps from -50 mV to various voltages in 10 sec intervals. A P/4 protocol was used for leak subtraction.

Index of Datasets S1 to S9

**Dataset S1**	Summary of Clinical Features of Sudden Unexplained Death in Childhood Cohort (N=124)
**Dataset S2**	Clinical Factors of Sudden Unexplained Death in Childhood Cohort (N=124)
**Dataset S3**	All de novo mutations called across Sudden Unexplained Death in Childhood Cohort
**Dataset S4**	All transmitted parental mosaic variants called across Sudden Unexplained Death in Childhood Cohort
**Dataset S5**	Membership of genes within the cardiac geneset (n=95) and the epilepsy geneset (n=43)
**Dataset S6**	Tests of significance of overlap of gene sets
**Dataset S7**	Overlap between disease gene sets and SUDC mutation gene set.
**Dataset S8**	Transmitted loss of function or ClinVar Pathogenic/Likely Pathogenic parental heterozygous variants in cardiac/epilepsy genes, called across SUDC Cohort
**Dataset S9**	Variants called across Sudden Unexplained Death in Childhood Cohort that make up pathogenic new genotypes

Supplementary Figures

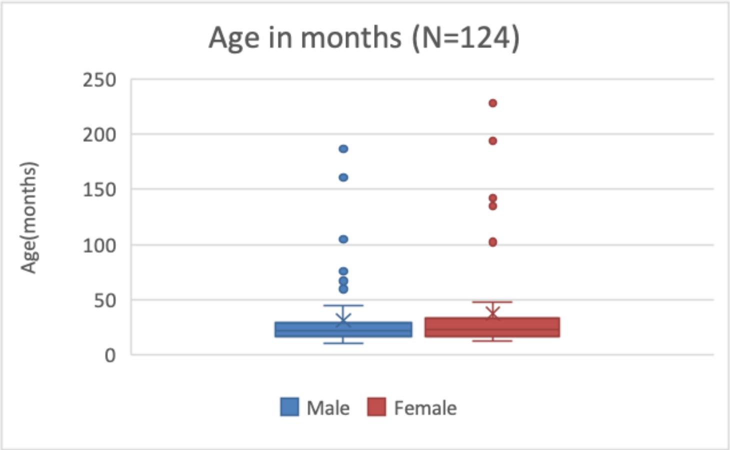

Fig. S1: Age at Death (N=124).

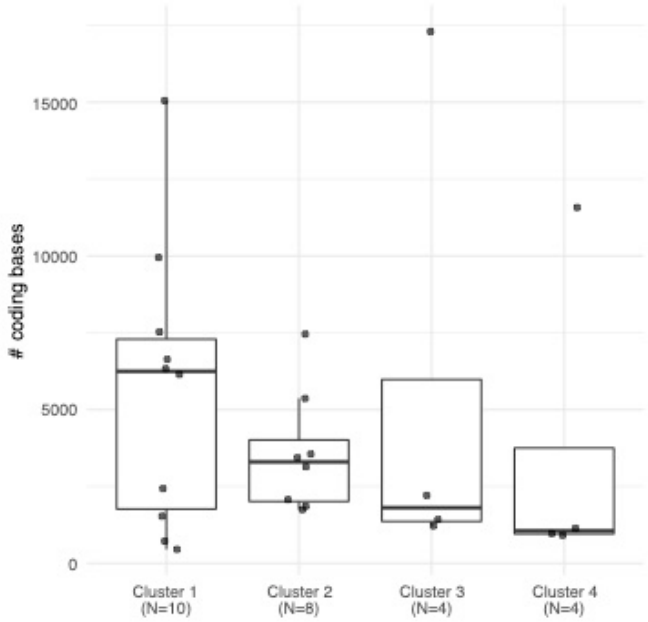

Fig. S2: Comparison of coding sequence sizes of genes identified in STRING PPI analysis.

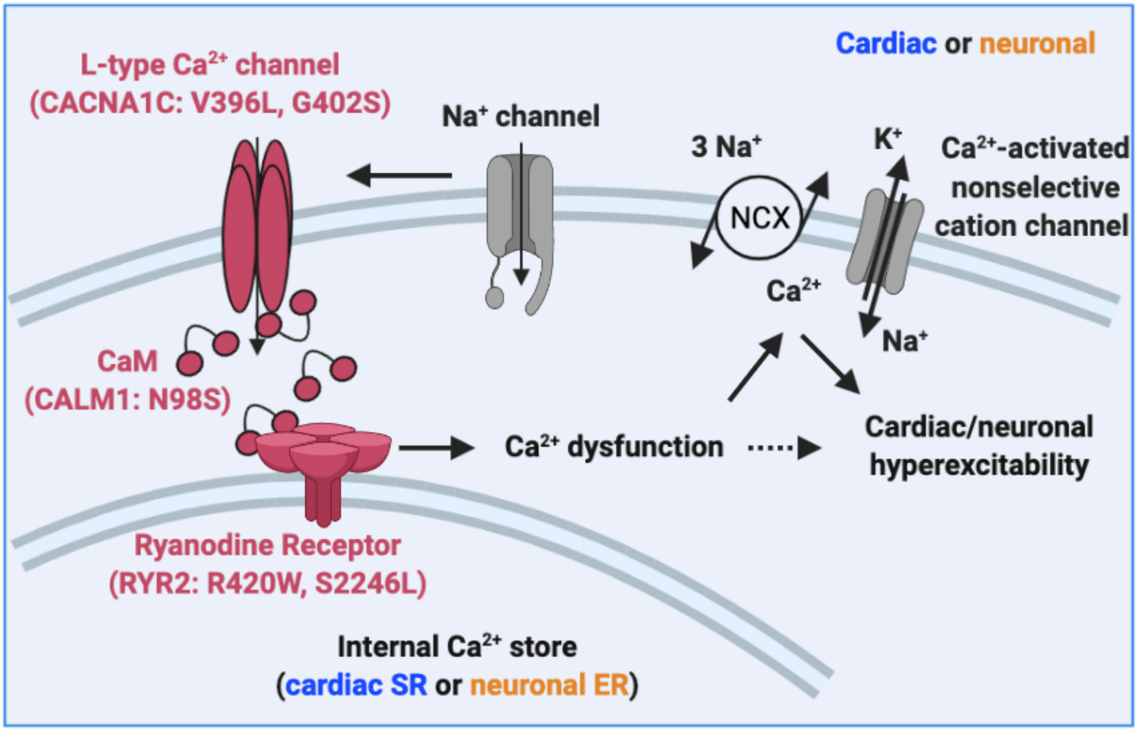

Fig. S3: Schematic summary of genes involved in calcium-regulation in cardiomyocytes or neurons that are implicated in SUDC pathogenesis. Six of the eight SUDC-associated *de novo* mutations identified in this study affect three genes (*CACNA1C*×2, *CALM1*, *RYR2*×2) that are involved in Ca^{2+} regulation at junctions between plasma membrane and internal Ca^{2+} stores (highlighted in red), and one that causes myofibrillary disarray (*TNNI3*), possibly affecting Ca^{2+} regulation (2). In turn, the Ca^{2+} dysfunction triggers inward sodium flux via various mechanisms and drives cardiac and/or neuronal hyperexcitability. SR, sarcoplasmic reticulum in cardiomyocytes; ER, endoplasmic reticulum in neurons.

SI References

1. M. Halvorsen *et al.*, Mosaic mutations in early-onset genetic diseases. *Genetics in medicine: official journal of the American College of Medical Genetics* **18**, 746 (2016).
2. H. Dridi *et al.*, "Intracellular calcium leak in heart failure and atrial fibrillation: a unifying mechanism and therapeutic target" in Nat Rev Cardiol. (England, 2020), vol. 17, pp. 732-747.
